# Continuous-flow electron spin resonance microfluidics device with sub-nanoliter sample volume

**DOI:** 10.1016/j.jmro.2025.100207

**Published:** 2025-07-09

**Authors:** Oleg Zgadzai, Nir Almog, Yefim Varshavsky, Moamen Jbara, Benoit Driesschaert, Aharon Blank

**Affiliations:** a Schulich Faculty of Chemistry Technion – Israel Institute of Technology Haifa, 3200003, Israel; b Department of Pharmaceutical Sciences, School of Pharmacy and In Vivo Multifunctional Magnetic Resonance Center, West Virginia University, Morgantown, WV 26506, USA

**Keywords:** ESR, Microfluidics, Micro resonators, Single cell measurements

## Abstract

This paper presents a novel continuous-flow electron spin resonance (ESR) microfluidic device designed for both continuous-wave (CW) and pulsed ESR measurements on sub-nanoliter liquid samples. The system integrates a planar surface microresonator (ParPar type) operating at ~9.4 GHz with a precision-fabricated quartz microfluidic chip, enabling spatial confinement of the sample within the resonator’s microwave magnetic field hotspot while minimizing dielectric losses. The effective sample volume is ~0.06 nL, and the device supports standard microfluidic connectors, facilitating both continuous and stopped-flow experiments. Using a 1 mM aqueous solution of deuterated Finland trityl (dFT) radical, CW ESR measurements yielded a peak signal-to-noise ratio (SNR) of ~83 for a 100-point spectrum acquired over 80 s, with a resonator quality factor of Q ~15–20. This corresponds to a spin sensitivity of ~1.04 × 10^9^ spins/√Hz/G. Pulsed ESR measurements, performed with 0.1 W microwave power and 10 ns π pulses, achieved an SNR of ~47 with 1 s of averaging, corresponding to a spin sensitivity of ~7.8 × 10^8^ spins/√Hz. A Rabi frequency of ~50 MHz was measured, indicating a microwave conversion efficiency of ~56 G/√W. Both the pulsed spin sensitivity and Rabi frequency are consistent with simulated values. This device represents a significant step toward ESR-based detection of individual, slowly flowing cells—analogous to flow cytometry but with magnetic resonance contrast. With future enhancements such as higher operating frequencies, cryogenic integration, or optimized resonator geometries, the system is expected to enable practical ESR measurements at the single-cell level.

## Introduction

Electron spin resonance (ESR) measurements of small-volume samples are essential when sample availability is limited. Examples include the study of biological molecules that are difficult to purify, unique isotopic compounds that are expensive to synthesize, or small single crystals that cannot be grown to large dimensions. In such cases, it is advisable to employ micro-resonators that enhance the absolute spin sensitivity of the ESR spectrometer. Several variants of micro-resonators have been reported in the literature. Some are more suitable for solid samples [1–5], while others can accommodate liquids or both types [6–11]. An important potential “add-on” to such resonators is a method for conveniently placing small-volume liquid samples onto or into the resonator—effectively creating an ESR microfluidic device. These devices have been described recently [10,12,13], enabling the measurement of small liquid volumes ranging from~0.25 nL [10] to ~30 nL [13] to 1 μL [12]. However, these setups generally do not support convenient continuous flow (or stopped flow) of the liquid sample.

The ability to continuously flow the sample over the resonator is critical for more advanced microfluidic experiments, particularly when the sample composition changes over time. One example is the study of chemical reactions that progress dynamically [14]. Another crucial application is the characterization and understanding of heterogeneity in cell populations at the *single-cell level*. Heterogeneity in cell populations is a pervasive phenomenon in all biological systems, including whole tissues and cultured cells, from bacteria to mammalian organisms. It is increasingly recognized that cell populations exhibit significantly more variability than previously assumed, and that bulk population analyses are inadequate to fully capture this complexity [15]. Accordingly, evaluating and interpreting cell-to-cell differences is now considered essential in biological research, and single-cell population analysis has become a key capability in modern biology and medicine. For instance, the ability to detect and analyze individual cells allows for deeper insights into disease origins, drug delivery mechanisms, and intercellular communication [16,17]. Single-cell analysis is not limited to biological metrics such as genomics, transcriptomics, and proteomics—it also extends to chemical and physical parameters. Thus, qualitative and quantitative chemical analysis at the single-cell level has attracted growing interest in the biological and medical sciences, offering new opportunities to investigate cellular function within complex environments [18,19].

It is therefore clear that single cells provide valuable models for in vivo processes. However, their use presents significant challenges, including their minute size, the limited amount of available material, and the need to process large numbers of individual samples at relatively high throughput. Progress in this area requires the development of advanced analytical techniques capable of quantitatively monitoring the chemical composition of minute samples during dynamic cellular events at the extracellular, whole-cell, and subcellular levels.

In this paper, we describe a new microfluidic ESR device primarily designed to enable future single-cell measurements as individual cells flow through the device. We begin by outlining the design, fabrication process, and electromagnetic properties of a specialized surface micro-resonator integrated with a superimposed glass microfluidic channel, developed specifically for this work. We then present both pulsed and continuous wave (CW) ESR experiments using deuterated Finland trityl (dFT) spin probe to evaluate the sensitivity of the setup. Finally, we discuss the potential of this approach to address key challenges in single-cell population analysis.

## The ESR microfluidic probehead

**Fig. 1.**
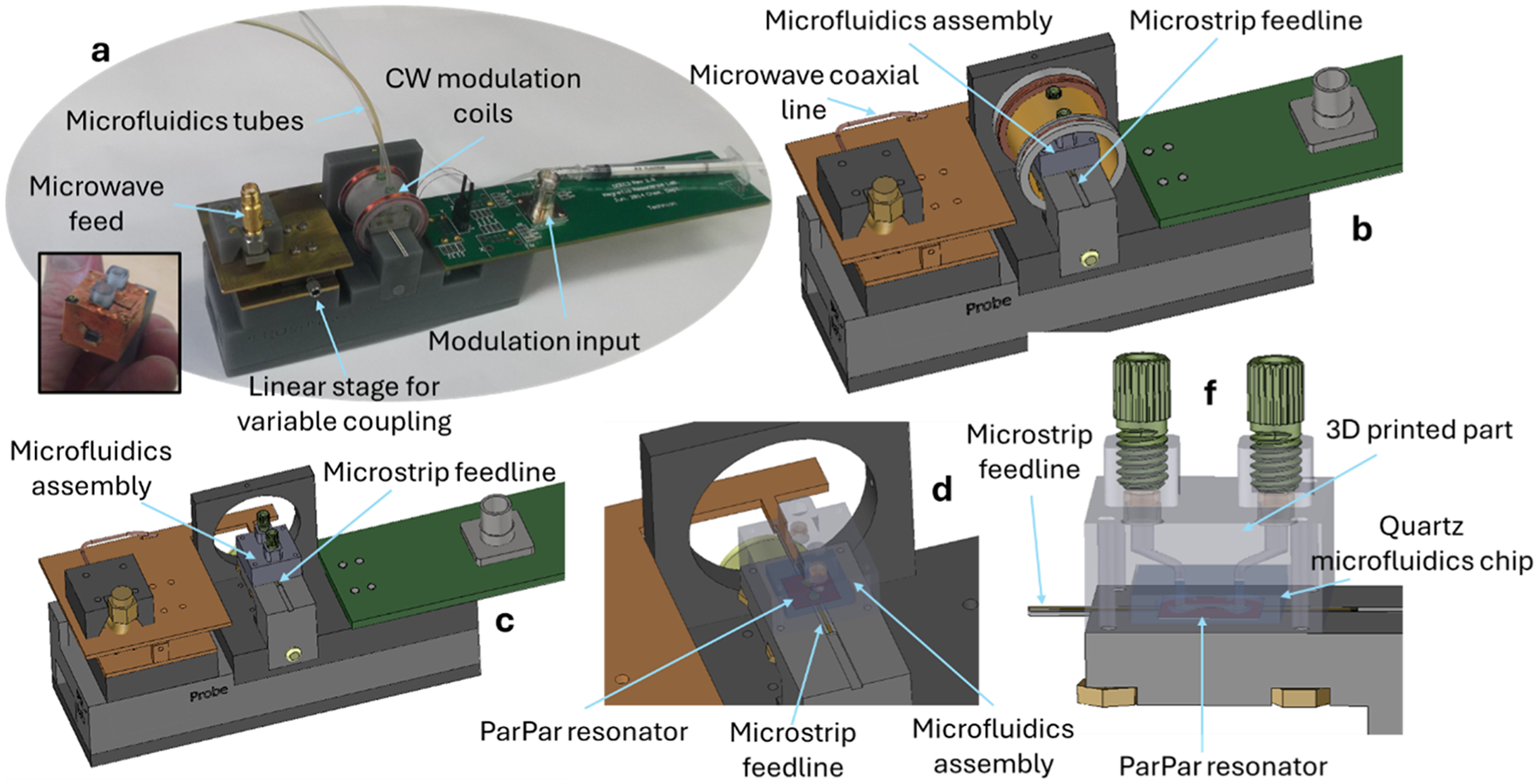
The ESR microfluidic probehead. (a) Photograph of the fully assembled probehead. (b) 3D rendering of the complete probehead assembly. (c) Same as (b), but with the magnetic field modulation coils removed to reveal the internal structure. (d) Close-up view of the central region, showing the microfluidic assembly in transparent mode. (e) Further zoom-in with a side view of the microfluidic assembly. The microwave signal is delivered from the SMA connector to the microstrip feedline via a custom-made coaxial-to-microstrip adapter. Microwave energy is coupled from the microstrip line to the resonator (and vice versa) positioned above it. This coupling is adjusted by changing the position of the end of the feedline relative to the resonator using a manual linear nonmagnetic stage (Elliot Scientific model MDE261A-LM). Optimal coupling is typically achieved when the end of the microstrip line is aligned just below the center of the resonator.

**Fig. 2.**
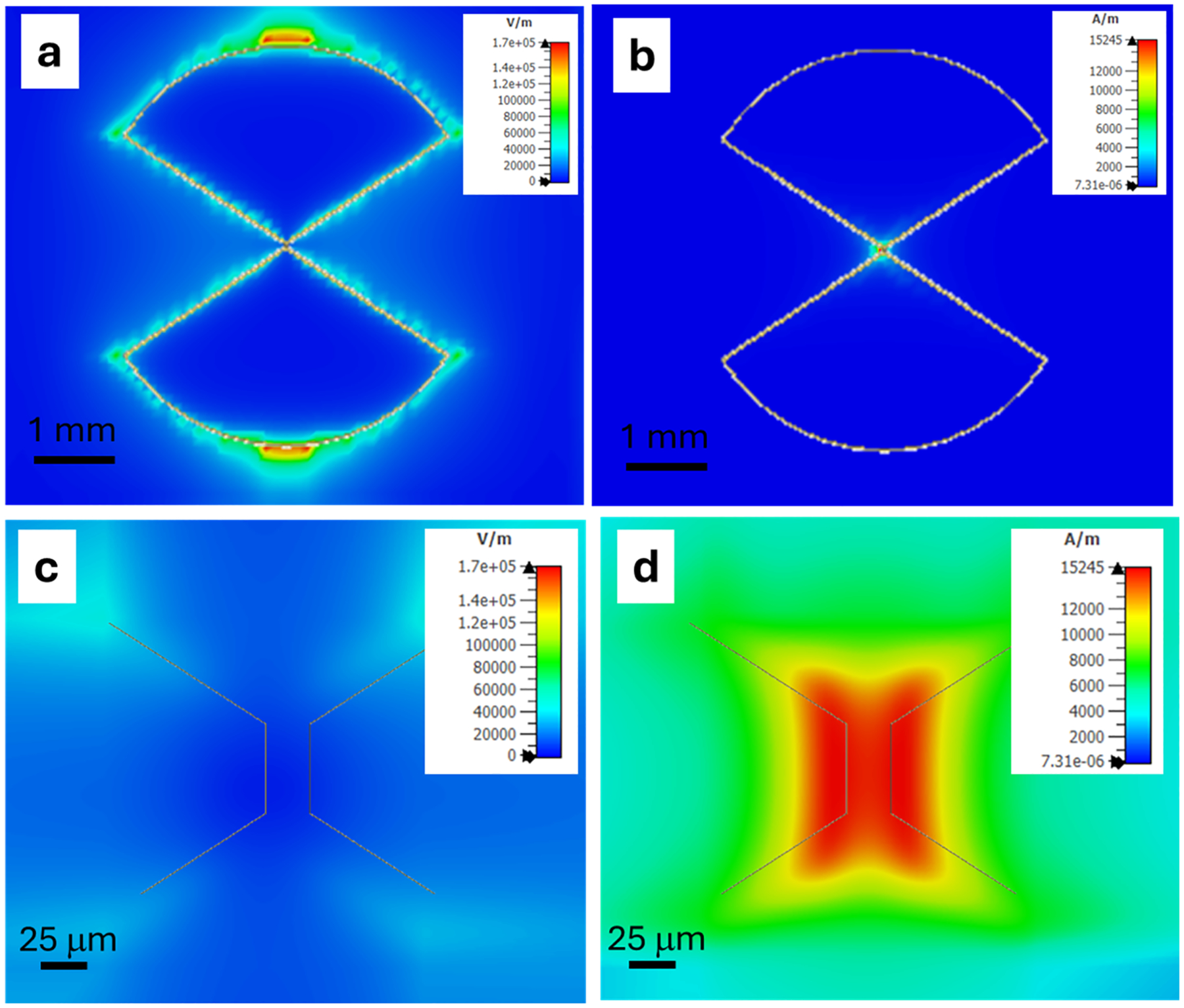
Surface microresonator used in the microfluidic ESR device. The resonator is fabricated by depositing 1 μm copper layer on 200 μm thick intrinsic silicon substrate [4,10]. Resonator diameter 4.63 mm. (a) Simulated microwave electric field (E-field) distribution at a height of 10 μm above the resonator surface for an input power of 1 W. (b) Simulated microwave magnetic field (H-field) distribution under the same conditions. (c) Magnified view of (a). (d) Magnified view of (b), showing that the H-field at the center of the resonator reaches approximately 15,000 A/m, corresponding to ~94.2 G/√W in the rotating frame, using the relation *B*_1_=*H*_1_ × 2π × 10^−7^ × 10^4^ (in Gauss). Simulations are carried out using CST Studio Suite finite element software (Dassault systems).

**Fig. 3.**
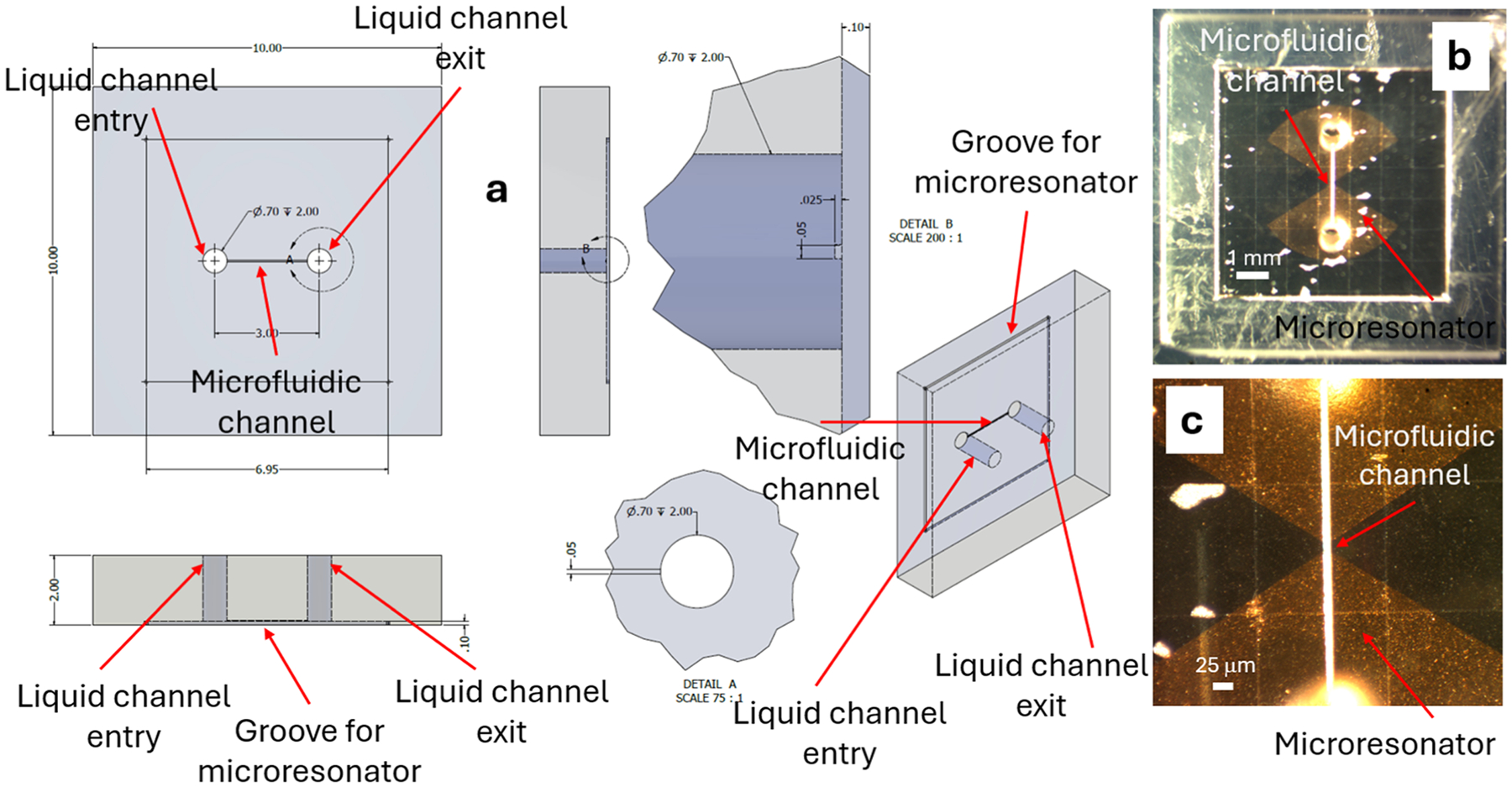
Quartz microfluidics chip. (a) Schematic drawing of the chip structure (dimensions in mm). (b) Microscope image of the chip glued to the surface microresonator, with the microfluidic channel precisely aligned over the ParPar resonator’s “bridge” region—characterized by low microwave electric field and high microwave magnetic field. (c) Magnified view of the central region in (b), showing the resonator center at higher optical resolution.

Fig. 1 presents the new probehead. At the heart of the system is a surface microresonator of the “ParPar” family, as shown in Fig. 1f [4]. For this application, we designed a microresonator operating at approximately 9–9.5 GHz (depending on the sample composition), which concentrates the microwave magnetic field in an area of ~50 × 50 μm (Fig. 2). Although the mode volume is small, the calculated quality factor (*Q*) of this resonator is relatively low—with an unloaded *Q* of 150 (or approximately 75 when critically coupled). The dominant loss mechanisms are metal deposition (*Q* ≈ 338) and radiation losses (*Q* ≈ 270), while dielectric losses from the silicon substrate are negligible (*Q* ≈ 1124). This resonator is bonded to a custom-designed quartz microfluidic chip fabricated using a high-precision 3D glass printer (LightFab, Germany), as illustrated in Fig. 3. The chip enables liquid flow through a narrow microfluidic channel measuring 50 μm in width and 25 μm in height, positioned directly above the resonator’s magnetic field hotspot (Fig. 3b–c). This configuration minimizes dielectric losses from the aqueous sample by reducing interaction with regions dominated by the microwave electric field (Fig. 2b).

In addition to the quartz microfluidic chip, a 3D-printed component (made of clear resin V4 by Formlabs 3D printer) is glued to the chip to complete the assembly. This part is coated with a thin copper foil (inset of Fig. 1a) to reduce radiation losses from the resonator. A key design feature is compatibility with industry-standard microfluidic connectors and 1/16″ OD Teflon tubing (models ID-M-644–03X, ID-M-650X, and BL-PTFE-1608–20 from Darwin Microfluidics). In our implementation, we used a 1 mL syringe (Fig. 1a) to manually drive liquid through the microfluidic channel, although any automated microfluidic system can be connected via the same tubing interface.

To support continuous-wave (CW) ESR measurements, we incorporated a pair of modulation coils, as shown in Fig. 1a–b. These coils have an inductance of approximately 294 μH, similar to that of the Bruker ER 4119HS X-band cavity used in our Bruker CW ESR system.

## Results

### Resonator microwave properties

**Fig. 4.**
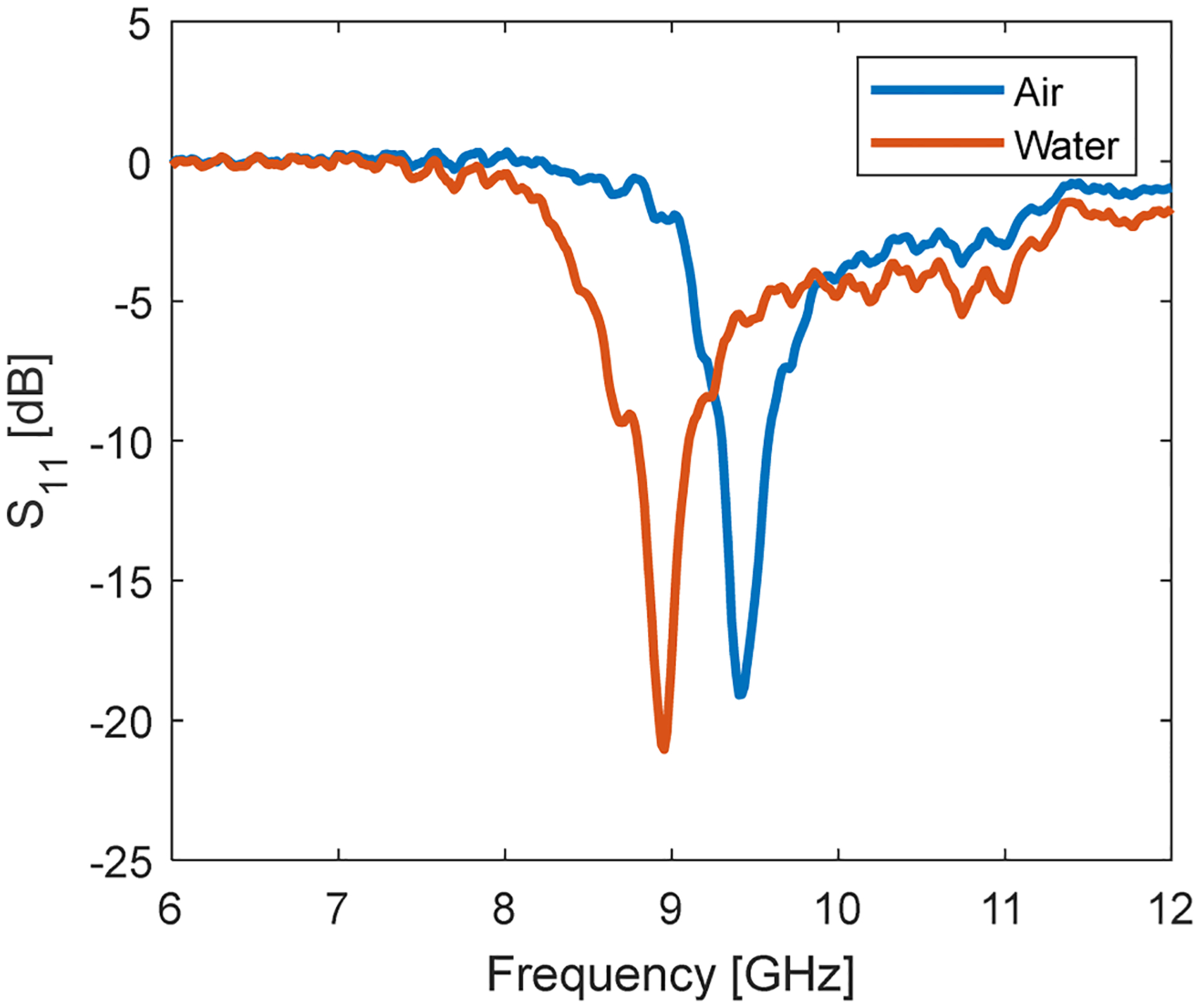
The reflection coefficient of the resonator as a function of frequency without any sample (blue) and when introducing the trityl water solution (red).

The microwave properties of the resonator were characterized using a vector network analyser. Fig. 4 shows the reflection coefficient (S_11_) of the resonator within the microfluidic probehead, measured both when the channel was empty and when filled with a 1 mM aqueous trityl solution. The quality factor (*Q*) of the resonator is typically evaluated by dividing the resonance frequency with the full width of the resonance dip at the −3 dB points. However, in our measurements, the resonance dip was asymmetric—likely due to imperfect microwave coupling. Combined with the relatively low *Q*, this asymmetry made it challenging to determine *Q* accurately using the standard method. To overcome this, we focused on the more regular (left) side of the S_11_ curve, where the −3 dB point could be more reliably identified. We estimated the half-width from this side and then extrapolated the full width of the resonance to calculate the *Q* factor. Using this approach, the loaded *Q* of the resonator (with an empty channel) was estimated to be approximately 15–20 at a resonance frequency of ~9.4 GHz. This is lower than the calculated values, probably due to the partial shielding vs. the calculated configuration. Introducing the lossy aqueous solution did not significantly degrade the *Q* factor, but it did shift the resonance frequency downward to approximately 9.0 GHz, likely due to changes in the effective dielectric environment of the resonator. The measured Q-factor correspond well to the predicted value from the electromagnetic simulation software.

### Results with continuous-wave (CW) ESR

**Fig. 5.**
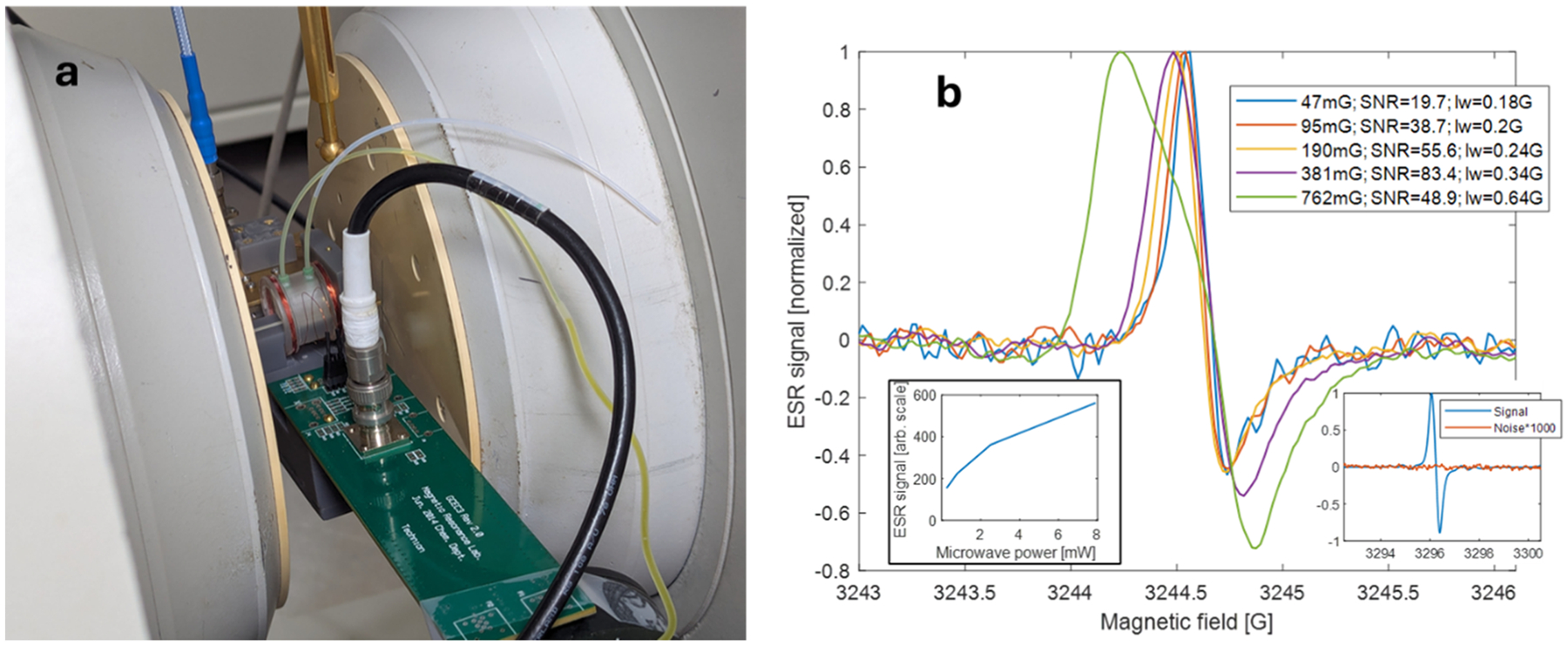
CW ESR measurements of a 1 mM dFT aqueous solution. (a) Photograph of the experimental setup showing the microfluidic ESR probe positioned inside the Bruker magnet, connected via coaxial (for microwave) and twinaxial (for field modulation) cables. (b) CW ESR spectra acquired at various magnetic field modulation amplitudes using 8 mW incident microwave power. Signal-to-noise ratio (SNR) and peak-to-peak linewidth (lw) values are indicated in the legend. The inset (lower left) shows the ESR signal intensity as a function of microwave power at a fixed modulation amplitude of 47 mG. Experimental parameters for all measurements: modulation frequency = 100 kHz; conversion time = 41 ms; integration time constant = 82 ms; 10 signal averages per acquisition. The inset (lower right) presents a reference measurement of the same radical sample placed in a 0.9 mm id glass capillary tube, performed under identical conditions of microwave power, conversion time, integration time, and field modulation, using a standard Bruker X-band cavity. The measured SNR in this case was approximately 5.4 × 10^4^, for a sample volume of 9.5 × 10^9^ μm^3^.

CW ESR measurements were performed using a Bruker EMX system operating at X-band. Fig. 5a shows the ESR microfluidic probe positioned inside the Bruker magnet, connected to the microwave bridge via a low-loss coaxial cable (following a waveguide-to-coaxial adapter at the microwave bridge output). The field modulation signal was delivered through a twinaxial cable connected directly to the probe using standard Bruker-compatible twinax connectors (female/male), identical to those used with conventional Bruker resonators (see Fig. 1a). Because the modulation coils in the probe are not identical to those in the standard Bruker cavity, we calibrated the modulation field values by comparing them to those indicated by the Bruker system software, using the expression provided on page 234 of ref [20].

Fig. 5b presents CW ESR spectra of a 1 mM aqueous solution of deuterated Finland trityl (dFT) radical—Tris(8-carboxyl-2,2,6,6-tetramethyl-(d_12_)-benzo-[1,2-d;4,5-d′]bis[1,3]dithiol-4-yl)methyl radical—synthesized according to a published procedure [21]. Spectra were acquired at varying magnetic field modulation amplitudes and incident microwave power levels, as indicated in the figure and its caption. Due to the low quality factor (*Q*) of the microresonator, the Bruker system’s automatic frequency control (AFC) was not effective. As a result, the recorded spectra exhibit a mixed absorption-dispersion lineshape. In addition, at power levels above ~10 mW, the reflected microwave power from the resonator became too high, leading to saturation of the detection diode signal—again due to the low *Q*. Nevertheless, the spectral features, including linewidths, are clearly resolved. The maximum signal-to-noise ratio (SNR), defined here as the peak signal amplitude relative to the RMS noise, reached 83.4 for a modulation amplitude of approximately 381 mG. Our results are also compared to those obtained using a standard Bruker X-band cavity to measure the same dFT sample (same modulation amplitude and microwave power). In that reference measurement, the sample was contained in a 0.9 mm capillary tube with a volume approximately 1.5 × 10⁵ times larger than that of our microfluidic device (Fig. 5b, right inset).

### Results with pulsed ESR

**Fig. 6.**
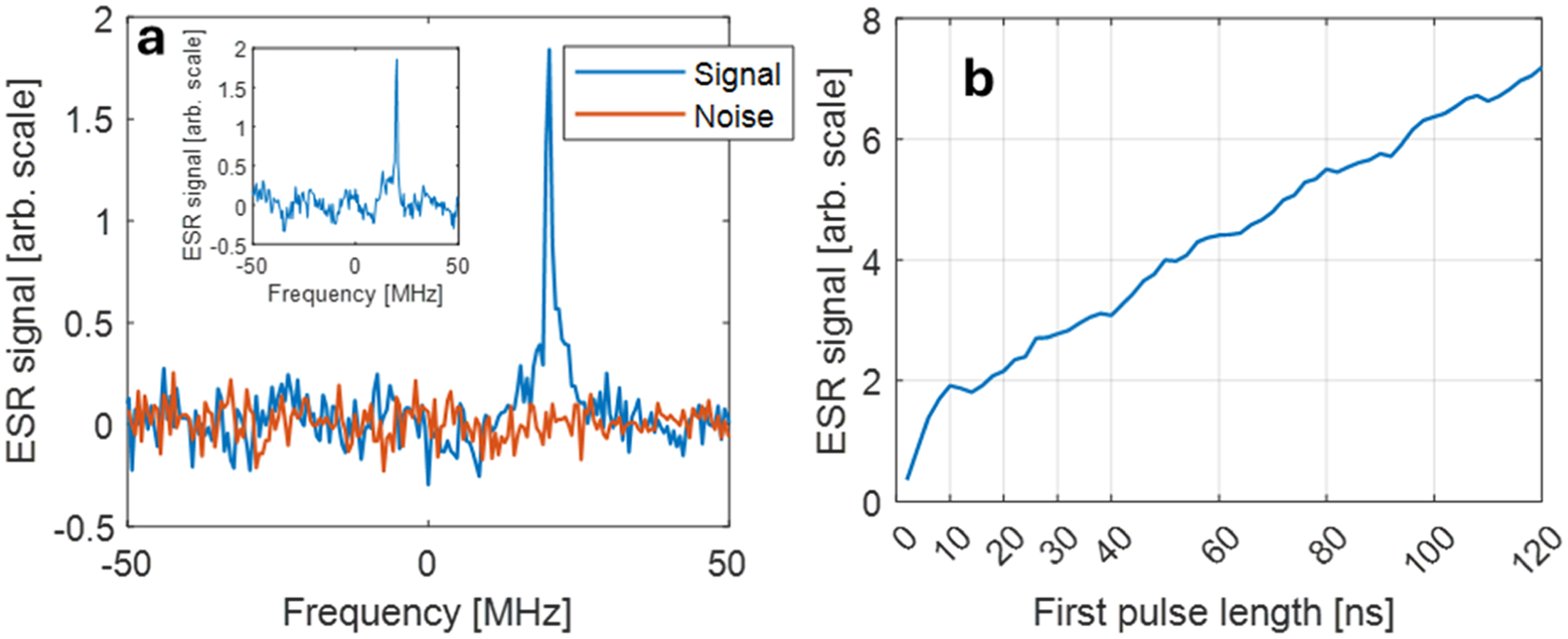
Pulsed ESR Hahn echo measurements of a 1 mM dFT aqueous solution. (a) Frequency-domain representation of the signal (blue) and noise (red, acquired 100 G off-resonance). Data were recorded using a 20 MHz off-resonance detection offset. Acquisition parameters: excitation frequency = 9.1 GHz; π/2 and π pulse durations = 10 ns; interpulse delay = 200 ns; microwave power = 0.1 W. The signal was averaged over 1 s at a repetition rate of 200,000 shots per second. An 8-step phase cycling scheme (+/− and CYCLOPS) was employed to suppress artifacts. (inset) The same experiment, carried out with 2 W of power and 2 ns-long pulses. (b) Rabi oscillation curve showing the ESR signal as a function of the duration of the first pulse in the Hahn echo sequence, with the second pulse fixed at 10 ns for 0.1 W of microwave excitation power.

Pulsed ESR experiments were performed using the SpinUp system (SpinFlex, Israel), operating over the 6–12 GHz frequency range. The same microresonator, microfluidic probe, and 1 mM aqueous solution of deuterated Finland trityl (dFT) radical used in the CW measurements were employed here. Fig. 6a displays the measured spin echo signal and corresponding noise, yielding a signal-to-noise ratio (SNR) of approximately 47 after one second of signal averaging. Although the microwave bridge can deliver up to 30 W of output power, the power was limited in this experiment to 0.1 W. This level was sufficient to produce a π pulse duration of 10 ns, as confirmed by the Rabi oscillation plot shown in Fig. 6b Based on this plot, the observed Rabi oscillation frequency at 0.1 W input power was found to ~50 MHz, which extrapolates to ~158 MHz at 1 W. This corresponds to a microwave magnetic field strength of approximately 56 G/√W, indicating good conversion efficiency of the resonator. To experimentally validate this scaling and ensure it is not merely a theoretical extrapolation, we performed an additional measurement at 2 W input power. In that case, a pulse duration of just 2 ns yielded an echo signal comparable in amplitude to that obtained with 0.1 W and 10 ns (see Fig. 6a, inset).

Additionally, we measured the spin–lattice (*T*_1_) and spin–spin (*T*_2_) relaxation times of the dFT solution using saturation recovery and Hahn echo pulse sequences, respectively. The measured values were found to be *T*_1_ = 527 ns and *T*_2_ = 442 ns (at 1 mM concentration, room temperature, and ambient atmospheric oxygen conditions).

## Discussion

The microfluidic ESR device presented in this work demonstrates the feasibility of performing both continuous-wave (CW) and pulsed ESR measurements on sub-nanoliter liquid samples under continuous or stopped-flow conditions. This represents a significant advancement in microscale ESR, particularly for applications requiring high sensitivity with minimal sample volumes—such as single-cell analysis or real-time reaction monitoring.

The integration of a surface microresonator with a precision-fabricated quartz microfluidic channel enables effective spatial confinement of the sample within the high microwave magnetic field region of the resonator, while minimizing dielectric losses. Despite the relatively low quality factor (*Q* ≈ 15–20), CW ESR measurements achieved a peak signal-to-noise ratio (SNR) of ~83 for a 100-point spectrum acquired in 80 s using a 1 mM aqueous dFT solution. This result confirms both the efficacy of the resonator design and the compatibility of the microfluidic system with standard CW ESR instrumentation. The effective sample volume is approximately 50 × 50 × 25 μm^3^ (~0.06 nL), defined by both the geometry of the microfluidic channel and the spatial distribution of the resonator’s magnetic field. For a 1 mM dFT solution, this corresponds to a total of ~3.7 × 10^10^ spins. Thus, for ~4.5 × 10^8^ spins a 100-point spectrum with an 80-second total acquisition time would yield an SNR = 1. This translates to a spin sensitivity of ~4.0 × 10^9^ spins for 1 second of acquisition, assuming similar spectral conditions for 100-point spectrum. Another common figure of merit in CW ESR is the spin sensitivity in units of spins/√Hz/G. In our case, we observed an SNR of ~83 for a modulation amplitude of ~0.38 G, using an effective averaging time of 800 ms (80 ms conversion time × 10 scans). This yields a spin sensitivity of approximately 3.7 × 10^10^/83 × √0.8/0.38 = 1.04 × 10^9^ spins/√Hz/G for the type of sample used in these experiments.

A comparison with the standard Bruker X-band CW ESR cavity (Fig. 5b, right inset) further highlights the performance of our microfluidic system. Under identical experimental conditions (modulation amplitude, microwave power, conversion and integration times), the Bruker cavity yielded an SNR of ~5.4 × 10^4^ using a 0.9 mm capillary with a sample volume ~1.5 × 10^5^ times larger than that of our microfluidic device. Despite this significantly larger volume, the increase in SNR was only by a factor of ~5.4 × 10^4^ / 83 ≈ 650. This implies that the absolute spin sensitivity of our ParPar-resonator-based microfluidic setup is approximately 1.5 × 10^5^/650~ 230 times better than that of the standard Bruker X-band cavity for CW ESR measurements.

Pulsed ESR experiments further validated the performance of the device under fast acquisition conditions. Using the same sample and resonator, Hahn echo measurements with only 0.1 W of microwave power yielded a signal-to-noise ratio (SNR) of ~47 after just 1 second of signal averaging. A π pulse duration of 10 ns corresponded to a Rabi frequency of ~50 MHz, indicating a microwave power conversion efficiency of *C*_p_ ~ 56 G/√W. This value agrees well with the simulated efficiency of ~94.2 G/√W at a position 10 μm above the resonator surface (Fig. 2d), when accounting for experimental losses and magnetic field non-uniformity. Assuming similar 80-second averaging conditions as used in the CW ESR experiments, the estimated spin sensitivity for pulsed ESR is approximately 8.8 × 10^7^ spins. This translates to a spin sensitivity of ~7.8 × 10^8^ spins for a 1-second acquisition (~7.8 × 10^8^ spins/√Hz) for this type of radical. These experimental results can be compared to theoretical predictions derived from the measured values of *C*_p_, *T*_1_, and the inhomogeneous ESR linewidth *T*_2_* ≈ 210 ns, using the expression provided in the Appendix of Ref. [22]. This yields a theoretical spin sensitivity of ~3.5 × 10^7^ spins/√Hz. The discrepancy between theory and experiment can largely be attributed to suboptimal experimental parameters. Specifically, the relatively long repetition time used in our experiment (5 μs) is significantly longer than the optimal value for the measured *T*_1_ of this radical (~1.25 × 527 = 658 ns), reducing the achievable SNR by a factor of √(0.6/5) ~ 0.34. Furthermore, signal loss due to spin–spin relaxation during the echo sequence (decay factor of (e^−420/*T*2^) ~ 0.38) contributes an additional reduction. Overall, these two effects account for roughly a 10-fold loss in sensitivity compared to the theoretical optimum, and once corrected, bring the experimental and theoretical values into good agreement.

One important point to note with respect to the data acquired in the pulse experiments is the unusual shape and features of the Rabi oscillation plot (Fig. 6b). The signal rises rapidly as the pulse length increases from 2 ns to 10 ns, then partially decays, followed by a steady increase for longer pulse durations. This behavior is consistent with the strongly inhomogeneous *B*_1_ field of the resonator and can be explained as follows: In the 2–10 ns regime, the signal originates primarily from the central “hot spot” above the bridge region of the resonator (Fig. 2b), where *B*_1_ is strongest. As the pulse length increases, signal contributions begin to arise from peripheral regions with much weaker *B*_1_ fields but much larger sample volumes—particularly near the capillary-glass–resonator interface. This explains the continuing increase in signal amplitude for longer pulses. The fact that the 10 ns pulse excites only the high- *B*_1_ central region confirms that this “hot spot” is the main contributor to the signal under short-pulse conditions.

In contrast, CW ESR lacks the possibility for spatial selectivity of pulsed excitation, making it more difficult to precisely define the excited sample volume. Therefore, caution should be taken when interpreting CW ESR sensitivity results unless the sample is strictly confined to the bridge region. Nevertheless, the good agreement between spin sensitivities measured by CW and pulsed ESR supports the conclusion that the dominant signal in both modalities originates from the same effective sample volume.

An important feature of the device is its compatibility with standard microfluidic systems. This enables precise control over the sample volume by administering the sample through the channel and monitoring the resonance frequency. In principle, flow can be halted once a frequency shift is detected, reducing the total sample required for single measurement to just ~0.1 nL. One promising application of this technology is the detection of slowly flowing cells—functionally analogous to flow cytometry, but with ESR readout. For example, we previously demonstrated that a trityl radical conjugated to a polyarginine cell-penetrating peptide can accumulate to concentrations of up to ~200 μM in MDA-MB-231 triple-negative breast cancer cells [23]. Given a typical cellular volume of ~2–6 pL [24], this corresponds to ~2.4 – 7.2 × 10^8^ spins per cell—well within the sensitivity range demonstrated here for a few tens of seconds of averaging time per cell.

Further improvements to the system are expected to enhance its performance. For example, reducing the dimensions of the resonator bridge and the microfluidic channel to ~10–25 μm, or transitioning to higher operating frequencies (e.g., ~35 GHz), could improve spin sensitivity by at least an order of magnitude. Another avenue for improvement is improving *Q* factor of the resonator through the reduction of radiation losses by introducing additional microwave shielding closer to the resonator surface in all directions. These enhancements could ultimately enable single-cell ESR measurements with averaging times on the order of seconds or less.

For CW ESR, signal quality could also be improved by implementing advanced signal processing techniques to extract pure absorption spectra, even in the absence of automatic frequency control (AFC) [25]. Another promising direction involves cryogenic integration of the microfluidic probe, enabling advanced ESR modalities such as DEER or low-temperature ENDOR for studying small-volume or single-cell systems.

Some limitations of the current setup should also be noted. The device lacks active temperature control, which could be critical for temperature-sensitive biochemical or biophysical assays. Additionally, fluid delivery was demonstrated using manual syringes; future integration with automated microfluidic pumps would improve throughput, reproducibility, and control.

In summary, the microfluidic ESR device described in this work represents a significant step forward in practical ESR microspectroscopy. It enables robust ESR measurements in flowing or stopped-flow systems and simplifies the administration of sub-nanoliter samples into surface microresonators. Its ease of integration with commercial ESR instruments, high sensitivity, and compatibility with microfluidic platforms establish it as a powerful tool for investigating dynamic chemical processes and enabling new single-cell ESR applications. Continued development of this technology could open new experimental regimes in both fundamental and applied electron spin resonance research.

## Data Availability

Data will be made available on request.
